# Association of CNR1 and INSIG2 polymorphisms with antipsychotics-induced weight gain: a prospective nested case–control study

**DOI:** 10.1038/s41598-021-94700-9

**Published:** 2021-07-27

**Authors:** Natalia Jimeno, Veronica Velasco-Gonzalez, Inmaculada Fierro, Mercedes Duran, Alfonso Carvajal

**Affiliations:** 1 Center for Drug Safety Studies (CESME), University of Valladolid, Ramon y Cajal Avenue, 7, 47005 Valladolid, Spain; 2 Pharmacogenetics, Cancer Genetics, Genetic Polymorphisms and Pharmacoepidemiology Research Group, University of Valladolid, Valladolid, Spain; 3 Department of Psychiatry, University of Valladolid, Valladolid, Spain; 4 Nursing Care Research Group (GICE), Department of Nursery, University of Valladolid, Valladolid, Spain; 5 Department of Health Sciences, Miguel de Cervantes European University, Valladolid, Spain; 6 Cancer Genetics Group, Institute of Genetics and Molecular Biology (UVa-CSIC), Valladolid, Spain

**Keywords:** Genetics, Diseases, Endocrinology, Health care

## Abstract

Weight gain is a frequent and severe adverse reaction in patients taking antipsychotics. The objective was to further investigate in a natural setting influential risk factors associated with clinically significant weight gain. An observational follow-up study was conducted. Patients when initiating treatment with whatever antipsychotic were included; a structured questionnaire was applied at baseline, 3 and 6 months later; a blood sample was obtained. In a nested case–control approach, patients with an increase ≥ 7% of their initial weight were considered as cases, the remaining, as controls. The results showed that, out of 185 patients, 137 completed the 6-month follow-up (cases, 38; controls, 99). Weight gain gradually and significantly increased in cases (baseline, 65.0 kg; 6 months, 74.0 kg) but not in controls (65.6 kg and 65.8 kg, respectively). Age (adjusted OR = 0.97, 95% CI = 0.96–0.99, *p* = 0.004), olanzapine (adjusted OR = 2.98, 95% CI = 1.13–7.80, *p* = 0.027) and quetiapine (adjusted OR = 0.25, 95% = 0.07–0.92, *p* = 0.037) significantly associated with weight gain. An association was also found for the *CNR1* (rs1049353) and *INSIG2* (rs7566605) polymorphisms. In conclusion, an increased risk of antipsychotics-induced weight gain was observed for younger age and olanzapine, and a relative lower risk for quetiapine. A potential role of *CNR1* rs1049353 and *INSIG2* rs7566605 polymorphisms is suggested.

Despite the severity and large number of their adverse reactions, antipsychotics are widely used in a variety of groups that include children and elderly people^1–3^; moreover, they are largely prescribed for indications other than schizophrenia^4^. Among the most troublesome adverse reactions are those related to metabolism, in particular weight gain^5–8^. Weight gain contributes to medication discontinuation and relapse; it also accounts for the metabolic syndrome and certainly increases morbidity and mortality^9^. In addition to environmental factors, genetics have been increasingly associated with weight gain when taking antipsychotics^10–13^; in particular, a twins study highlighted the significance of genetics at this regard^14^.

Numerous clinical trials have been published on patients treated with antipsychotics^15^; however, prospective follow-up studies carried out in real-life settings are scanty. Hence, the objective of this study was to further investigate influential risk factors as associated with significant weight gain in a natural setting.

## Material and methods

### General

For this purpose, an observational prospective follow-up study was conducted and, for the analysis, a nested case–control approach was adopted. Patients willing to participate, 14 years old and older, when initiating treatment with whatever antipsychotic and with a body mass index (BMI) lower than 35 kg/m^2^, were included; a washout period of at least 6 months was needed for those previously on antipsychotics. The recruitment period was March 2010 to December 2014; patients were recruited in hospitals, in community services or in nursing homes.

After obtaining a written informed consent, trained monitors directly interviewed patients with a structured questionnaire; the questionnaire was filled at baseline and then prospectively at 3 and 6 months after recruitment. Information upon the following types and specific variables was collected: (i) social and demographic (age, sex, education, working status); (ii) metabolic diseases ([yes/no]); (iii) psychiatric (diagnoses, type of diagnosis [psychotic/non-psychotic disorder], number of psychiatric diagnoses); (iv) substance use (alcohol intake [yes/no]); smoking ([yes/no]); (v) all medications (for antipsychotics: type of drug, number and dose in chlorpromazine equivalence); and (vi) anthropometrics (height [cm], body weight [kg], and BMI [kg/m^2^]; height and body weight were assessed using a SECA 217 height rod and a SECA 877 scale, respectively; vii) activity factor^16^. Indirectly, Charlson index was calculated^17^.

### Genotyping

In addition, a venous blood sample (9 ml) was obtained from study participants at the first visit for genetic analysis. Based on results of published studies as well as a search in the PubMed database (http://www.ncbi.nlm.nih.gov/PubMed), relevant candidate genes previously associated with antipsychotics-induced weight gain were selected. In this manner, a total of 38 polymorphisms in 14 candidate genes were selected for the present study: Ankyrin repeat and kinase domain containing 1 (*ANKK1*): rs1800497, rs7104979, rs17115461; Brain-derived neurotrophic factor (*BDNF*): rs6265; Cannabinoid receptor 1 (*CNR1*): rs1049353; Dopamine receptor D2 (*DRD2*): rs1799978, rs1799732; Fatty acid amide hydrolase (*FAAH*): rs324420; Fat mass and obesity associated (*FTO*): rs76804286, rs9926289, rs9939609; Guanine nucleotide binding protein (*G protein*), beta polypeptide 3 (*GNB3*): rs5442, rs5443; 5-hydroxytryptamine (serotonin) receptor 2A (*HTR2A*): rs6313, rs1805055; 5-hydroxytryptamine (serotonin) receptor 2C (*HTR2C*): rs6318, rs518147, rs1414334, rs5946226, rs3813928, rs3813929; Insulin induced gene 2 (*INSIG2*): rs7566605, rs10490624, rs11889497, rs17047764, rs17587100; Leptin (*LEP*): rs791615, rs75550733, rs7798338, rs7799039; Leptin receptor (*LEPR*): rs1137101; Melanocortin 4 receptor (*MC4R*): rs489693, rs2229616, rs8087522, rs11872992, rs17066842, rs17782313; Peroxisome proliferator activated receptor gamma (*PPARG*): rs1801282.

Purified DNAs were quantified by Picogreen. The amplicons were prepared using the Access Array System for Illumina Platform (Fluidigm). Briefly, this method consist of the following phases: (1) quantification by Picogreen and normalization of the DNA samples to 50 ng/ul, (2) combination of diluted DNA samples with primers (5′-AATGATACGGCGACCACCGAGATCTACACTGACGACATGGTTCTACA-3′ and 5′-CAAGCAGAAGACGGCATACGAGAT-[barcode]-TACGGTAGCAGAGACTTGGTCT-3′) of the Access Array Barcode Library for Illumina Sequencers (Fluidigm), (3) preparation of 20X specific target primers solutions, (4) loading of sample inlets and primer inlets of a primed Access Array Integrated Fluidic Circuit (IFC) and running of the PCR AA48 × 48 Standard v1 protocol and, finally, (5) harvest of PCR products from the IFC. For more details consult the protocol: “Access Array System for Illumina Platform User Guide_G1”.

The finally obtained amplicons were validated and quantified by an Agilent 2100 Bioanalyzer using High Sensitivity chips, and an equimolecular pool of these amplicons was purified by agarose gel electrophoresis to eliminate primers/dimers, and titrated by quantitative PCR using the “Kapa-SYBR FAST qPCR kit forLightCycler480” and a reference standard for quantification. The pool of amplicons was denatured prior to be seeded on a flowcell at a density of 10 pM, where clusters were formed and sequenced using a “MiSeq Reagent Kit v3”, in a 2 × 300 pair-end sequencing run on a MiSeq sequencer. Raw sequences were filtered according to quality under standard Illumina, parameters and positive sequences were mapped against human genome. Following mapping, DNA variant detection was performed using the MiSeqReporter software (Illumina).

### Statistics analysis

For the case–control approach, patients who, at 180 days, showed an increase ≥ 7% of their initial weight were considered as cases^18^; the remaining were considered as controls. Values of qualitative variables are presented as absolute frequencies and percentages; quantitative variables are presented as means with Standard Deviation (SD) or median with interquartile ranges [Q1–Q3]. For comparison among qualitative variables, including Hardy–Weinberg equilibrium, the Pearson’s chi-square test, or the Fisher’s exact test if the expected frequency was lower than 5 in any cell, were used. Genotypes distribution in the two groups in comparison was assessed with the Monte Carlo method for multiple testing^19^; the estimated *p* values in this form are in Table 3; for comparison among groups for quantitative variables, a Student t test or a Mann–Whitney U were calculated according to the Kolmogorov–Smirnov or Shapiro–Wilk test. To explore possible correlation between some quantitative variables without normal distribution, Spearman´s Rho was calculated.

Based on the literature, covariates initially considered for adjusting in the analysis were: age, sex, BMI at baseline and the different SNPs considered. Information on exposure to the explanatory variables was that consigned at baseline. Other possible confounders were selected among those with a *p* < 0.1 significance level in a previous univariate analysis; a forward stepwise multivariate logistic regression was performed with those variables. With the significant variables in this first regression, a second regression analysis was performed, minimizing in this way the possible effect of the missing values in variables which finally were non-significant. A Hosmer–Lemeshow goodness-of-fit test was performed. To learn the influence of each of the several factors on weight gain, adjusted odds ratios (adjusted OR) and their 95% confidence intervals (95% CI) were calculated. For all tests, significance level was set at *p* < 0.05. The Statistical Package for Social Sciences (version 24.0; SPSS, Inc., Chicago, IL) was used for statistical analyses.

### Ethics

The protocol of the study was approved by the Ethics and Clinical Research Committee of the Faculty of Medicine, University of Valladolid, Spain (CEIC); it was included in the Spanish and in the European Medicines Agencies registers. The study was conducted following the principles of the Declaration of Helsinki and all applicable local ethical and legal requirements. All participants provided written informed consent before performance of the study procedures.

### Results

**Figure 1:**
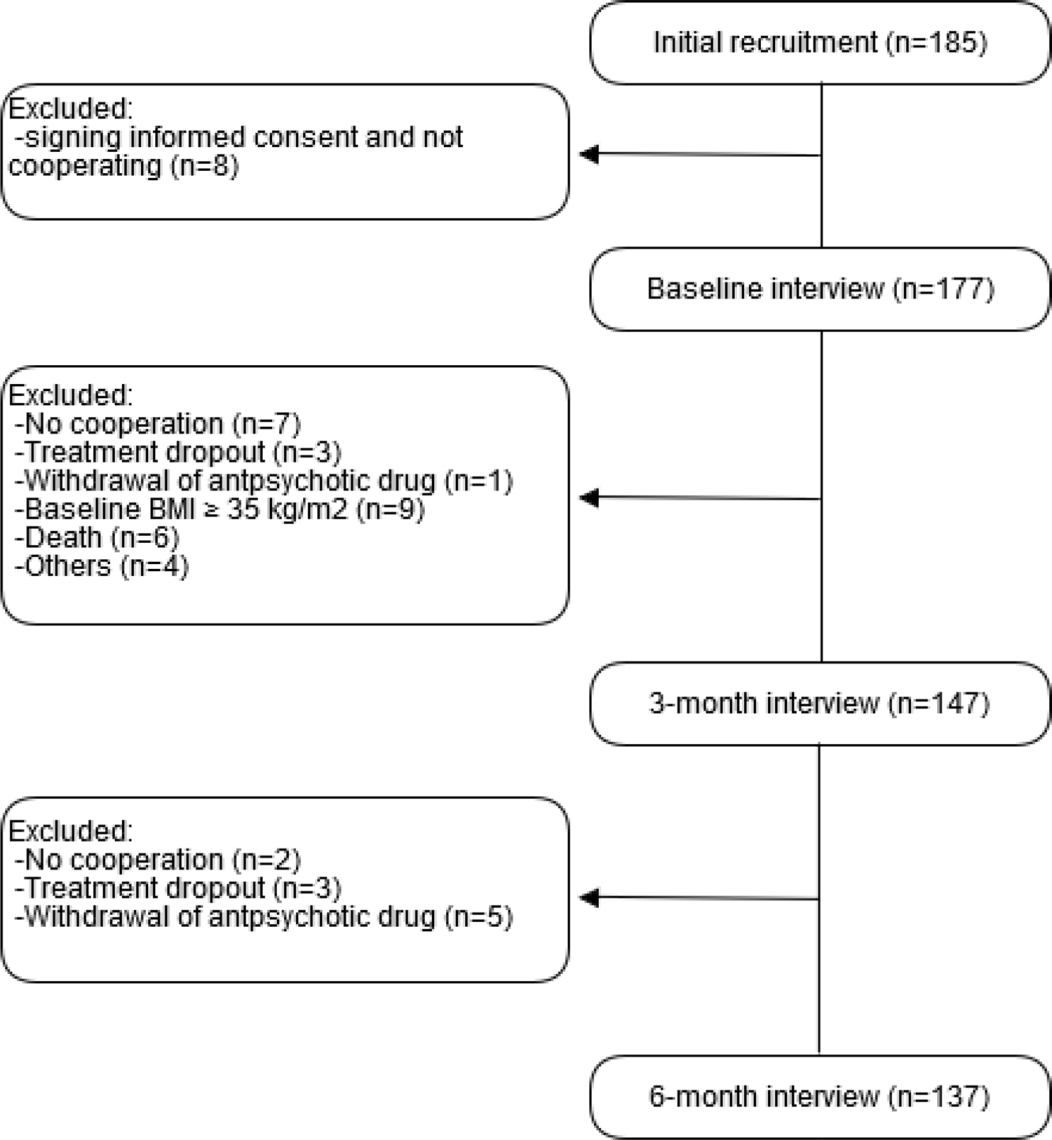
Flowchart of the patients’ follow-up. Main baseline characteristics of those patients who fulfil inclusion criteria but were lost to follow-up (n = 39): age, 56.2 years (55.2 years in the final cohort); mean BMI, 23.8 kg/m^2^ (25.5 in the final cohort); females, 44.7% (63.5% in the final cohort). They did not substantially differ from those who were finally included.

Out of 185 patients of the ongoing cohort, 137 completed at least a follow-up period of 6 months and were included in the current analysis; reasons for dropouts are presented in a flow-chart (Fig. 1). Of those patients who completed the specified period, 38 showed a clinically significant increase of their initial weight and were accordingly considered as cases; the remaining 99, who did not, were considered as controls.

The whole sample was of European origin. The distribution of the main sociodemographic and clinical variables in the two groups is presented in Table 1. Mean age in the whole sample was 55.2 years, it ranged from 15 to 100 years; age was higher in controls than in cases (59.7 years vs 43.4 years, *p* = 0.001). Though there were more female patients in the cohort (63.5%), no significant differences between cases and controls were found (*p* = 0.654). As an average, patients had 1.1 psychiatric diagnoses and were having 1.2 antipsychotics; risperidone, followed by quetiapine, olanzapine and aripiprazole were the antipsychotics most frequently used. More cases than controls received two or more antipsychotics during some time in the follow-up period (34.2% vs. 20.2%, *p* = 0.086); cases received higher doses than controls (median chlorpromazine equivalence, 237.74 mg/d vs. 117.24 mg/d, respectively; *p* = 0.002). More psychotic diagnoses were observed within the cases without statistically significant difference (39.5% vs. 24.2%, *p* = 0.08). Previous history of metabolic disease was associated with a minor risk of weight gain (*p* = 0.018); similarly, higher Charlson index scores were associated with less risk (*p* = 0.008; Table 1). No differences in overall medications were found between cases and controls (*p* = 0.072).

**Table 1:** Distribution of the main characteristics in the two groups in comparison.

	Cases (n = 38)	Controls (n = 99)
	n (%)	n (%)
Mean age, y (SD)*	43.39 (20.72)	59.66 (24.23)
< 35 years	14 (36.8)	18 (18.2)
35–55 years	18 (47.4)	28 (28.3)
> 55 years	6 (15.8)	53 (53.5)
**Sex**		
Male	15 (39.5)	35 (35.4)
Female	23 (60.5)	64 (64.6)
Initial body weight mean, kg (SD)	65.05 (12.71)	65.65 (14.49)
Education*^a^		
Basic	18 (48.6)	66 (71.0)
Higher	19 (51.4)	27 (29.0)
**Psychiatric diagnoses**^**b**^		
Psychotic disorders	15 (39.5)	24 (24.2)
Non-psychotic disorders	23 (60.5)	75 (75.8)
**Number of psychiatric diagnoses**		
One	34 (89.5)	88 (88.9)
More than one	4 (10.5)	11 (11.1)
**Antipsychotics**		
Risperidone	18 (47.4)	46 (46.5)
Quetiapine*	3 (7.9)	27 (27.3)
Olanzapine*	13 (34.2)	12 (12.1)
Aripiprazole*	11 (28.9)	12 (12.1)
Paliperidone	4 (10.5)	8 (8.1)
Haloperidol	3 (7.9)	8 (8.1)
Others^c^	3 (7.8)	8 (8.0)
**Number of antipsychotics**		
One	25 (65.8)	79 (79.8)
More than one	13 (34.2)	20 (20.2)
Chlorpromazine equivalence, median mg/d [IQR]	237.74 [117.05–476.17]	117.24 [45.42–295.30]
Charlson index*		
Absence of comorbidity	33 (86.8)	63 (63.6)
Presence of comorbidity^d^	5 (13.2)	36 (36.4)
Median of overall medications, [IQR]^e^	5.0 [3.0–8.0]	6.0 [4.0–11.0]
Metabolic history*	5 (13.2)	33 (33.3)
Activity factor, median [IQR]	1.37 [1.33–1.44]	1.36 [1.33–1.41]
Alcohol intake	10 (26.3)	14 (14.1)
Smoking*^f^	22 (57.9)	28 (28.3)

*p < 0.05.

^a^No data, 7 patients (1 case, 6 controls); Basic education: reading and writing, primary and secondary education.

^b^Psychiatric diagnoses were made by medical specialists. Psychotic disorders cover schizophrenia, schizotypal, delusional, and other non-mood psychotic disorders (F20 to F29 codes). Non-psychotic disorders cover mental disorders due to known physiological conditions (F00 to F09 codes), mental and behavioral disorders due to psychoactive substance use (F10 to F19 codes), mood [affective] disorders (F30 to F39 codes) and other mental disorders (F40 to F99 codes), according to the International Classification of Diseases, 10th edition.

^c^Amisulpride, asenapine, sulpiride, tiapride, ziprasidone.

^d^Include both low and high comorbidity.

^e^Medications influencing somehow weight gain were metformin (cases, 0; controls, 5), levothyroxine (cases, 3; controls, 6), topiramate (cases, 3; controls, 5), digoxin (cases, 1; controls, 2) and methylphenidate (cases, 0; controls, 2). Information for these drugs is provided in the Summary of Product Characteristics.

^f^No tobacco abstinence cases were registered.

**Figure 2:**
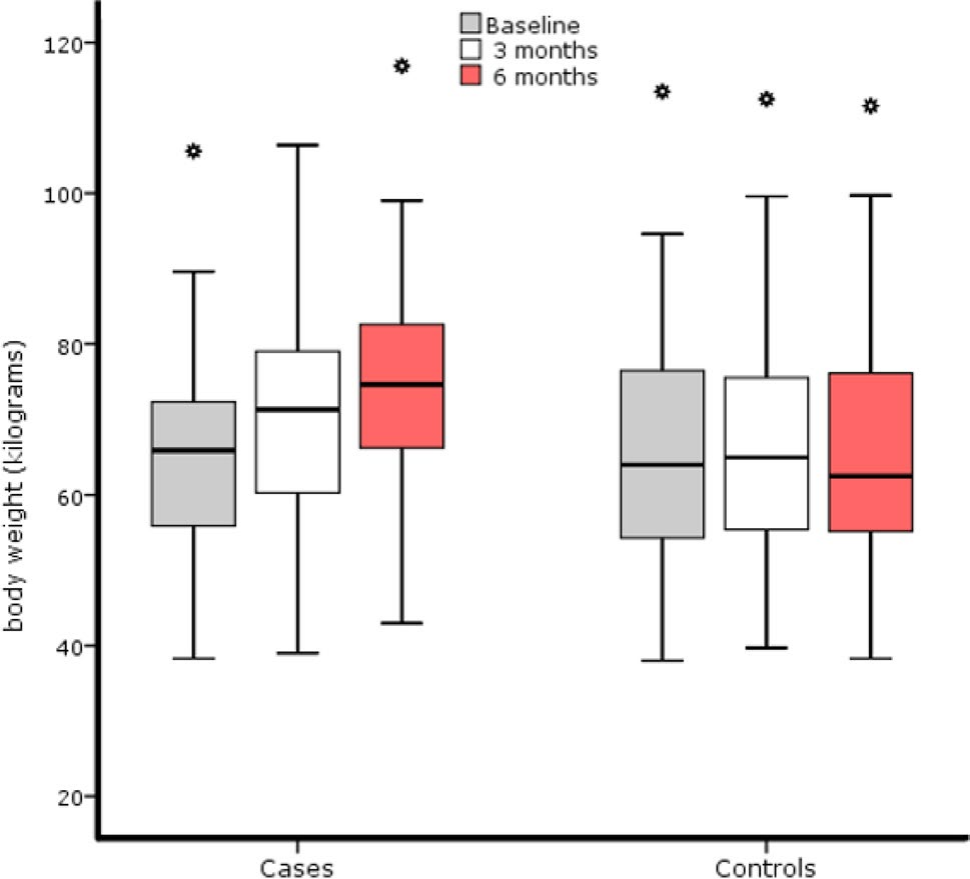
Body weight evolution by study groups.

Cases were taller than controls (mean values (SD), 1.64 (0.10) m vs. 1.59 (0.12) m, respectively; *p* = 0.01); body weight was similar in the two groups at baseline (65.05 (12.71) kg vs. 65.65 (14.49) kg, respectively; *p* = 0.824), but it differed at the end of the follow-up (73.97 (14.33) kg vs. 65.84 (14.97) kg for cases and controls, respectively; *p* = 0.003). The average weight increase was 8.92 (4.08) kg for cases and 0.19 (2.99) kg for controls. Weight gradually increased in cases but not in controls (Fig. 2). Regarding BMI, this was different at baseline (cases, 24.23 (4.07) kg/m^2^; controls, 26.00 (4.24) kg/m^2^; *p* = 0.029) but it equalized at the end of follow-up (cases, 27.51 (4.48) kg/m^2^; controls, 26.07 (4.27) kg/m^2^; *p* = 0.082). No differences in physical activity existed between cases and controls, neither at baseline (*p* = 0.379) nor at the end of the follow-up period (*p* = 0.608) when measured by the activity factor (Table 1).

In our multivariate model only age, along with exposure to olanzapine or quetiapine, showed a significant association with weight gain (Table 2). Sex was not significant in the model, not even for olanzapine or quetiapine exposure as an interaction term. Dose was not significant either; when taken independently a significant correlation was observed between dose in chlorpromazine equivalents and percentage of weight gain (Rho = 0.31; *p* = 0.0003). A lower risk of weight gain was observed with increasing patients’ age and for those treated with quetiapine, this similarly occurs when choosing an age range of 18–65; risk of weight gain significantly increased for patients who were treated with olanzapine.

**Table 2:** Risk factors for antipsychotic-induced weight gain.

	Cases (n = 38)	Controls (n = 99)	Crude OR (95% CI)	*p*	Adjusted OR (95% CI)	*p*
Age^a^	38 [24–54]	58 [39–83]	0.97 (0.95–0.99)	0.001	0.97 (0.96–0.99)	0.004
Olanzapine^b^	13 (34.2)	12 (12.1)	3.77 (1.53–9.29)	0.004	2.98 (1.13–7.80)	0.027
Quetiapine^b^	3 (7.9)	26 (26.3)	0.23 (0.07–0.81)	0.022	0.25 (0.07–0.92)	0.037

^a^Median [IQR].

^b^As compared with the other antipsychotics.

When examined by a 2 × 3 table Pearson’s chi-square test (two degrees of freedom), a trend of significant differences between subjects who gained 7%, or higher, of their baseline body weight, and those who did not, emerged for *CNR1* (rs1049353) and *INSIG2* (rs7566605) polymorphisms (Tables 3 and 4); *CNR1* (rs1049353) was associated with weight change in a dominant model (OR = 0.41, 95% CI = 0.18–0.94, *p* = 0.034) while *INSIG2* (rs7566605) was in a recessive model (OR = 3.03, 95% CI = 1.05–8.78, *p* = 0.041). After adjusting by age and medication effects, adjusted OR were respectively (OR = 0.50, 95% CI = 0.21–1.21, *p* = 0.13) and (OR = 2.17, 95% CI = 0.67–6.99, *p* = 0.195).

**Table 3:** Genotypic and allelic frequencies of certain polymorphisms in patients treated with antipsychotics.

Genes	Polymorphism	Chromosome	Major allele	Genotype	Total n (%)	Cases n (%)	Controls n (%)	*p*-value^a^	HWE^b^ *p*-value
ANKK1	rs1800497	11	T	TT CT CC	6 (4.4) 39 (28.5) 92 (67.2)	1 (2.6) 12 (31.6) 25 (65.8)	5 (5.1) 27 (27.3) 67 (67.7)	0.792	0.307
	rs7104979	11	C	CC CT	134 (97.8) 3 (2.2)	37 (97.4) 1 (2.6)	97 (98.0) 2 (2.0)	1.000	0.919
	rs17115461	11	G	AA	137 (100.0)	38 (100.0)	99 (100.0)	–	
BDNF	rs6265	11	G	GG GA AA	87 (64.0) 44 (32.4) 5 (3.7)	26 (70.3) 11 (29.7) 0 (0.0)	61 (61.6) 33 (33.3) 5 (5.1)	0.403	0.845
CNR1	rs1049353	6	C	CC CT TT	81 (59.1) 52 (38.0) 4 (2.9)	28 (73.7) 10 (26.3) 0 (0.0)	53 (53.5) 42 (42.4) 4 (4.0)	0.092	0.218
DRD2	rs1799732^c^	11	A	AA T-/TG TG-TG TG/TG	8 (5.8) 22 (16.1) 105 (76.6) 2 (1.5)	4 (10.5) 5 (13.2) 28 (73.7) 1 (2.6)	4 (4.0) 17 (17.2) 77 (77.8) 1 (1.0)	–	
	rs1799978	11	G	GG AG AA	1 (0.7) 13 (9.5) 123 (89.8)	0 (0.0) 3 (7.9) 35 (92.1)	1 (1.0) 10 (10.1) 88 (88.9)	1.000	0.261
FAAH	rs324420	1	C	CC CA AA	89 (66.9) 38 (28.6) 6 (4.5)	25 (67.6) 10 (27.0) 2 (5.4)	64 (66.7) 28 (29.3) 4 (4.2)	0.946	0.675
FTO	rs9926289	16	A	AA GA GG	26 (19.0) 52 (38.0) 59 (43.1)	5 (13.2) 18 (47.4) 15 (39.5)	21 (21.2) 34 (34.3) 44 (44.4)	0.325	0.006
	rs9939609	16	A	AA TA TT	26 (19.0) 52 (38.0) 59 (43.1)	5 (13.5) 18 (47.4) 15 (39.5)	21 (21.2) 34 (34.3) 44 (44.4)	0.325	0.006
	rs76804286	16	G	GG GA	135 (98.5) 2 (1.5)	37 (97.4) 1 (2.6)	98 (99.0) 1 (1.0)	0.479	0.960
GNB3	rs5442	12	G	GG GA AA	123 (89.8) 13 (9.5) 1 (0.7)	33 (86.8) 5 (13.2) 0 (0.0)	90 (90.9) 8 (8.1) 1 (1.0)	0.655	0.117
	rs5443	12	C	CC CT TT	53 (38.7) 61 (44.5) 23 (16.8)	13 (34.2) 17 (44.7) 8 (21.1)	40 (40.4) 44 (44.4) 15 (15.2)	0.653	0.615
HTR2A	rs6313	13	C	CC CT TT	37 (27.0) 71 (51.8) 29 (21.2)	7 (18.4) 21 (55.3) 10 (26.3)	30 (30.3) 50 (50.5) 19 (19.2)	0.312	0.821
	rs1805055	13	C	CC CT	138 (98.5) 2 (1.5)	38 (100.0) 0 (0.0)	97 (98.0) 2 (2.0)	1.000	0.919
HTR2C^d^	rs6318	X	G	GG GC CC	113 (82.5) 17 (12.4) 7 (5.1)	31 (81.6) 4 (10.5) 3 (7.9)	82 (82.8) 13 (13.1) 4 (4.0)	0.589	0.884
	rs518147	X	G	GG GC CC	74 (54.0) 31 (22.6) 32 (23.4)	22 (57.9) 8 (21.1) 8 (21.1)	52 (52.5) 23 (23.2) 24 (24.2)	0.906	0.224
	rs1414334	X	C	CC CG GG	13 (9.5) 18 (13.1) 106 (77.4)	6 (15.8) 4 (10.5) 28 (73.7)	7 (7.1) 14 (14.1) 78 (78.8)	0.327	1.000
	rs3813928	X	G	GG GA AA	101 (73.7) 20 (14.6) 16 (11.7)	28 (73.7) 8 (21.1) 2 (5.3)	73 (73.7) 12 (12.1) 14 (14.1)	0.207	0.021
	rs3813929	X	C	CC CT TT	101 (73.7) 20 (14.6) 16 (11.7)	28 (73.7) 8 (21.1) 2 (5.3)	73 (73.7) 12 (12.1) 14 (14.1)	0.207	0.021
	rs5946226	X	C	CC CT TT	13 (9.5) 18 (13.1) 106 (77.4)	6 (15.8) 4 (10.5) 28 (73.7)	7 (7.1) 14 (14.1) 78 (78.8)	0.331	1.000
INSIG2	rs7566605	2	G	GG CG CC	60 (43.8) 61 (44.5) 16 (11.7)	18 (47.4) 12 (31.6) 8 (21.1)	42 (42.4) 49 (49.5) 8 (8.1)	0.048	0.224
	rs10490624	2	A	AA AG GG	107 (78.1) 29 (21.2) 1 (0.7)	28 (73.7) 9 (23.7) 1 (2.6)	79 (79.8) 20 (20.2) 0 (0.0)	0.257	0.264
	rs11889497	2	T	TT TC	118 (83.9) 22 (16.1)	33 (86.8) 5 (13.2)	82 (82.8) 17 (17.2)	0.795	0.350
	rs17047764	2	C	CC GC GG	2 (1.5) 40 (29.2) 95 (69.3)	2 (5.3) 10 (26.3) 26 (68.4)	0 (0.0) 30 (30.3) 69 (69.7)	0.104	0.076
	rs17587100	2	A	AA AG	120 (87.6) 17 (12.4)	30 (78.9) 8 (21.1)	90 (90.9) 9 (9.1)	0.081	0.636
LEP	rs791615	7	G	GG AA	133 (97.1) 4 (2.9)	36 (94.7) 2 (5.3)	97 (98.0) 2 (2.0)	0.308	0.919
	rs7798338	7	A	AA AG	136 (99.3) 1 (0.7)	38 (100.0) 0 (0.0)	98 (99.0) 1 (1.0)	1.000	0.960
	rs7799039	7	G	GG GA AA	51 (37.2) 66 (48.2) 20 (14.6)	10 (26.3) 21(55.3) 7 (18.4)	41 (41.4) 45 (45.5) 13 (13.1)	0.243	0.906
	rs72550733	7	G	GG GA	135 (98.5) 2 (1.5)	38 (100.0) 0 (2.0)	97 (98.0) 2 (2.0)	1.000	0.919
LEPR	rs1137101	1	A	AA AG GG	60 (43.8) 50 (36.5) 27 (19.7)	15 (39.5) 14 (36.8) 9 (23.7)	45 (45.5) 36 (36.4) 18 (18.2)	0.730	0.033
MC4R	rs489693	18	A	AA GA GG	9 (6.6) 23 (16.8) 105 (76.6)	2 (5.3) 5 (13.2) 31 (81.6)	7 (7.1) 18 (18.2) 74 (74.7)	0.791	0.001
	rs2229616	18	G	GG GA	133 (97.1) 4 (2.9)	38 (100.0) 0 (0.0)	95 (96.0) 4 (4.0)	0.576	0.837
	rs8087522	18	A	AA GA GG	10 (7.3) 51 (37.2) 76 (55.5)	3 (7.9) 17 (44.7) 18 (47.4)	7 (7.1) 34 (34.3) 58 (58.6)	0.485	0.518
	rs11872992	18	G	GG GA AA	101 (73.7) 28 (20.4) 8 (5.8)	29 (76.3) 7 (18.4) 2 (5.3)	72 (72.7) 21 (21.2) 6 (6.1)	0.942	0.019
	rs17066842	18	A	GG GA	127 (92.7) 10 (7.3)	36 (94.7) 2 (5.3)	91 (91.9) 8 (8.1)	0.726	0.675
	rs17782313	18	T	TT TC CC	94 (68.6) 38 (27.7) 5 (3.6)	27 (71.1) 9 (23.7) 2 (5.3)	67 (67.7) 29 (29.3) 3 (3.0)	0.674	0.948
PPARG	rs1801282	3	C	CC CG GG	111 (81.0) 24 (17.5) 2 (1.5)	29 (76.3) 9 (23.7) 0 (0.0)	82 (82.8) 15 (15.2) 2 (2.0)	0.453	0.207

^a^*p*-value (2-sided) Monte Carlo method (10.000 samples) for 2 × 3 tables or Fisher exact test for 2 × 2 tables.

^b^Hardy-Weinberg equilibrium (HWE) for the control group.

^c^An indel SNP was detected (rs1799732).

^d^For the genotype association analysis of the variants in this gene, males have been considered as homozygous females which precludes Hardy–Weinberg equilibrium. HWE was calculated only for women.

**Table 4:** Genotypic and allelic frequencies of the *CNR1* (rs1049353) and *INSIG2* (rs7566605) polymorphisms associated with weight gain in patients treated with antipsychotics.

Gene (SNP)	Our MAF	HapMap MAF	Cases	Controls	OR (95% CI)	*p*-value
CNR1 (rs1049353)						
Genotype						
* CC*			28	53	1.0 (ref.)	
* CT*			10	42	0.45 (0.20–1.03)	0.059
* TT*			0	4	–	–
Dominant genotype model						
*CT TT* vs *CC*			10/28	46/53	0.41 (0.18–0.94)	0.034
Alleles						
*C*			66	148		
*T*	21.9%	25.2%	10	50	0.45 (0.21–0.94)	0.034
INSIG2 (rs7566605)						
Genotype						
* GG*			18	42	1.0 (ref.)	
* CG*			12	49	0.57 (0.25–1.32)	0.191
* CC*			8	8	2.33 (0.76–7.19)	0.140
Recessive genotype model						
*CC* vs *CG GG*			8/30	8/91	3.03 (1.05–8.78)	0.041
Alleles						
*G*			48	133		
*C*	33.9%	29.4%	28	65	1.94 (0.69–2.07)	0.570

## Discussion

Our observational study following a nested case–control approach showed that one in three patients on antipsychotics developed an increase of ≥ 7% of their initial weight after a period of 6 months (cases) when compared with those who did not (controls). This significant and severe weight gain associated with antipsychotics has been largely observed elsewhere: at 6 weeks in 45.8% of schizophrenic patients on olanzapine^20^ and in 20.2% of bipolar patients with the same medication^21^; at 3 months in 35% of schizophrenic first-episode medication-naive patients^22^. In a large systematic review and meta-analysis, a significantly higher risk of gaining ≥ 7% of the baseline weight (RR = 2.04, 95% CI = 1.54–2.71, *p* < 0.001) was also found after 3 to 12 weeks of treatment, and the most severe weight-gain was caused by olanzapine^5^. Weight gain also has been found in longer follow-up studies at 6, 12 and 36 months^23–25^. Our figures are in this range; follow-up duration, antipsychotics used, psychiatric disease, comorbidities and, particularly, age certainly account for these differences in percentages. Taking as a reference the sample studied by Verma et al.^24^, with a similar 6-month period of observation, it can be observed that differences in percentages of patients who gained 7% or more than the baseline weight (ours, 27.7%; Verma's, 65%) inversely correlated with average age differences in the two samples (ours, 55.1 years; Verma´s, 29.8 years).

Although percentage of male are generally higher in schizophrenia samples using antipsychotics, female patients are majority in our sample; this is probably due to the more frequent non-psychotic disorders in women, including neurocognitive and affective disorders. The influence of sex in antipsychotic-induced weight gain is still controversial; moreover, sex did not appear to emerge as a critical predictive factor for beneficial effect^26^. We did not observe differences in weight gain between sexes, what is coincident with previous observations by Zipursky et al.^27^. Though most of the studies found a higher risk in females^1,24,28–30^ and lower in males in the first months of treatment^23^, the figures by Zipursky et al.^27^ remain chiefly reliable because they are based on a survival analysis at 2 years in a study specifically designed to find out predictors of weight gain in people with first-episode psychosis.

As far as age is concerned, younger age seems to be consistently associated with a higher risk of weight gain associated with antipsychotics^28,30,31^; being children and adolescents even at a greater risk^32,33^. In fact, younger age, along with non-Caucasian ethnicity, low baseline BMI, along with other variables, are considered one of the 16 items for Weight Gain Risk Factor Assessment Checklist^30^. In our sample, a higher proportion of patients younger than 35 years showed an increase of weight (43.7%) compared to those of 35–55 years (39.1%), or those older than 55 years (10.2%). According to our estimates, for each year of increasing age, the risk of weight gain diminishes 3%; this estimate remained stable, barely without changes from the crude estimate. Thus, increasing age appears as a protective factor for antipsychotics-associated weight gain in the studied sample. This has been consistently observed in other studies^24,29,31,34^; it must be underlined the susceptibility observed in children and adolescents to antipsychotics-associated obesity^32,35,36^. Age is also associated with certain diseases, comorbidities, and the corresponding treatments: i.e., doses of antipsychotics in elderly populations are usually lower than those used in the youngest ones. Moreover, age is related to physiological changes, which could be related to a less propensity for weight gain. None of these factors seem to have a significant modifying effect in our estimate.

Up to eleven different antipsychotics were used by patients in our sample (Table 1); all, but haloperidol, were atypical. Olanzapine was independently and strongly associated with weight gain in our sample (Table 2); this is consistent with previous findings^1,21,23,31,37–39^ and with two meta-analyses, where olanzapine showed the highest risk of weight gain^5,40^. Although in our study quetiapine increased the initial weight in 0.4 kg as an average, this increase was lower than that observed with the other antipsychotics; this is coincidental with results of a recent meta-analysis^5^. Most of the patients in the cohort received only one psychiatric diagnosis and had one antipsychotic, though receiving several antipsychotics is a common practice in schizophrenia^41,42^; this lower use found might be related to the lower proportion of psychotic patients in our sample.

In this study exploring 38 polymorphisms located in 14 candidate genes, two were found to be associated with antipsychotics-induced weight gain in *CNR1* and *INSIG2* genes (Table 3). The minor T-allele of the frequently studied synonymous variant in the final exon of *CNR1* (rs1049353) conferred some protection; it was observed that carriers of the minor allele gained less weight while treated with antipsychotics than the CC homozygotes (OR = 0.41, 95% CI = 0.18–0.94, *p* = 0.034). To our knowledge, four studies have explored this association so far^43–46^. Our results are coincidental with those found by Nurmi et al.^43^ in a relatively large sample of children with autism treated for the first time with antipsychotics (n = 181); they differ, however, from the results found by the others. In the study by Monteleone et al.^44^, carried out similarly in a Mediterranean population like ours (MAF, 17% and 22%, respectively), comparable associations would be expected; notwithstanding, subtle differences in design might account for the disparities observed: i.e., patients who had been previously exposed to antipsychotics being already obese could participate in that study (n = 83). Regarding the study by Park et al*.*^45^ (n = 78), the differences in allelic distribution of the Asian population they analyzed, with a MAF of 7%, by far lower than ours (22%), make it difficult to detect any association when the exposure in the cases is low, as it was the case (6.7%). The study by Tiwari et al*.*^46^ in samples exposed to clozapine and olanzapine with an elevated initial body weight (n = 183) neither found any association in a shorter follow-up period of 14 weeks, though it was found with other polymorphisms in the same gene; all in all, it is concluded that the *CNR1* gene may be associated with antipsychotics-induced weight gain in chronic schizophrenia. As for the *INSIG2* polymorphism (rs7566605), we found an association with weight gain in a recessive model. A genome-wide study by Skelly et al*.*^47^ looked at the same marker in a large sample of patients treated with antipsychotics from the CATIE study^1^; for the same recessive model, it was observed an analogous trend in magnitude to that first reported by Herbert et al.^48^ in the general population [in subjects of European ancestry, mean BMI (SD) was 31.9 (0.94) for the CC genotype versus 30.8 (0.43) for combined CG/GG genotypes]. As denoted by these figures, a possible explanation for these inconclusive results might be the elevated baseline BMIs of the patients included in the CATIE study^1^; otherwise, a study primarily intended to assess a different outcome. Also, the study by Tiwari et al*.*^49^ found certain association for the same allele with this variant in a subset of patients with African ancestry who received either clozapine or olanzapine (n = 54). Conversely, two studies exploring the same variant did not find any statistically significant relationship (n = 160 and n = 128, respectively)^50,51^; patients in both studies, with shorter durations than ours, had been previously exposed to antipsychotics, being stated in one of the studies that it could bias the identified amount of body weight gain^51^.

The cannabinoid receptor (CNR1 or CB1) is related to food intake and lipogenesis^52^. *Cnr1* knockout mice presents a lean phenotype^53–55^, and, consistently, administration of the inverse agonist rimonabant leads to decreased body weight^56,57^; on the contrary, endogenous ligands of CNR1 increases dietary food intake^58^. Insulin induced gene 2 protein (INSIG2) on its part inhibits cholesterol synthesis in the adipose tissue and the liver^59^; dysregulation of this process may produce obesity and increase insulin resistance. Both *CNR1* and *INSIG2* are involved in the processing of sterol regulatory element binding proteins (SREBPs): CNR1 receptor activity leads to increased lipogenesis through augmented expression of these SREB proteins^60,61^ and, on the contrary, INSIG2 blocks these proteins acting as a negative regulator of cholesterol biosynthesis^62^. Hence, it is conceivable that variants in genes encoding these proteins may alter their normal functioning, accounting for metabolic dysregulation and yielding to weight gain; this is what has been observed in our study: protection with a *CNR1* variant and risk with other *INSIG2* variant.

Among the study limitations, sample size and heterogeneity are the most remarkable. Heterogeneity is linked to natural settings in which patients are quite different in diagnoses, ages, and treatments; thus, it makes it difficult to identify minor risk factors since larger samples are needed for stratification. Conversely, this setting and the observational nature of our study provides the actual variety of patients on these medications; in addition, a close follow-up has permitted a more detailed collection of the main features of all individuals in the study which includes adherence—dose collected were those that patients were taking. As for genetic association analysis, the stringent inclusion criteria upon use of antipsychotics—most of our patients were drug naive prior to the beginning of the study limiting the influence of one of the most confounding factors in previous studies—and weight along with the prospective character of this study make it suitable to clearly observe the development of the condition studied and assess the genetic risk factors; though, in terms of genetic association studies, the number of subjects is rather small, it still represents a comparably large sample for the specific phenotype. Hidden population always remains as a possible confounder; however, all individuals in our sample were from European origin, they come from the same geographical region, Castile (Spain), without cultural differences. No migrants or adopted children were found in the recruited patients; moreover, the Spanish population is generally like that of Northern and Western Europe origin, but also largely homogeneous within itself^63,64^.

In summary, our study has identified two major risk factors for weight gain when on antipsychotics, younger age and olanzapine, as well as a third relative protective factor, quetiapine; these influential factors are fully discussed in the light of more information. Additionally, the observations made in the large screening of this study indicate a possible role of certain polymorphisms in *CNR1* and *INSIG2* genes in antipsychotic-induced weight gain; at this regard, new approaches in the study design considering the likely polygenic character of the genetics influence for weight gain should be adopted. All in all, our results should be interpreted as exploratory and require replication in an independent and larger data set.
